# Hervey virus: Study on co-circulation with Henipaviruses in Pteropid bats within their distribution range from Australia to Africa

**DOI:** 10.1371/journal.pone.0191933

**Published:** 2018-02-01

**Authors:** Claudia Kohl, Mary Tachedjian, Shawn Todd, Paul Monaghan, Victoria Boyd, Glenn A. Marsh, Gary Crameri, Hume Field, Andreas Kurth, Ina Smith, Lin-Fa Wang

**Affiliations:** 1 Robert Koch Institute, Centre for Biological Threats and Special Pathogens, Seestraße 10, Berlin, Germany; 2 CSIRO Health and Biosecurity, Australian Animal Health Laboratory, Geelong, Victoria, Australia; 3 Queensland Centre for Emerging Infectious Diseases, Department of Agriculture and Fisheries, Brisbane, Queensland, Australia; 4 EcoHealth Alliance, New York, United States of America; 5 Programme in Emerging Infectious Diseases, Duke-NUS Medical School, Singapore, Singapore; University of Pretoria, SOUTH AFRICA

## Abstract

In 2011, an unusually large number of independent Hendra virus outbreaks were recorded on horse properties in Queensland and New South Wales, Australia. Urine from bat colonies adjacent to the outbreak sites were sampled and screened for Hendra and other viruses. Several novel paramyxoviruses were also isolated at different locations. Here one of the novel viruses, named Hervey virus (HerPV), is fully characterized by genome sequencing, annotation, phylogeny and *in vitro* host range, and its serological cross-reactivity and neutralization patterns are examined. HerPV may have ecological and spatial and temporal patterns similar to Hendra virus and could serve as a sentinel virus for the surveillance of this highly pathogenic virus. The suitability of HerPV as potential sentinel virus is further assessed by determining the serological prevalence of HerPV antibodies in fruit-eating bats from Australia, Indonesia, Papua New Guinea, Tanzania and the Gulf of Guinea, indicating the presence of similar viruses in regions beyond the Australian border.

## Introduction

Over recent decades, bats have been recognized to play a major role in the emergence of infectious diseases [1–5]. Their role as reservoir hosts for the highly pathogenic order *Mononegavirales*, such as filoviruses, lyssaviruses and henipaviruses, has led to increased interest in bats, the oldest known mammals on earth [6].

*Henipavirus* is a genus within the family *Paramyxoviridae* that consists of Hendra, Nipah and Cedar viruses which have been isolated from pteropid bats and humans in Asia or Australia [3, 4, 7]. The family *Paramyxoviridae* consists of seven genera (*Aquaparamyxovirus*, *Avulavirus*, *Ferlavirus*, *Henipavirus*, *Morbillivirus*, *Respirovirus* and *Rubulavirus*) [8]. Viruses from the various genera have been detected in bat tissues and body fluids throughout Africa, Central and South America, Europe, Asia and Australia, and co-circulation was often assumed [9–18]. Though many paramyxoviruses have been detected by PCR, very rarely have live viruses been successfully isolated. The high number of reports on paramyxoviruses of bats is underlining the possibility of a long co-evolution between bats and paramyxoviruses, explaining the high diversity and co-circulation of paramyxoviruses found in bats.

Hendra virus outbreaks occur on a regular basis in Australia, and the 55 distinct outbreaks have claimed the lives of four humans and 97 horses between 1994 and 2016. In 2011, the highest annual number of Hendra virus outbreaks was reported with 18 individual events [12, 19]. The reasons for the unusually high number of outbreaks in 2011 are not fully understood. It is presumed that the potential drivers of these increases could be anthropogenic in nature. Prior to the 2011 outbreaks, large areas of Queensland (QLD) were exposed to significant weather events (i.e., severe tropical cyclone Yasi in February 2011), altering the normal feeding behaviour of the reservoir hosts, flying foxes. A number of research groups have hypothesized that such events could increase stress in the bats and lead to increased shedding of viruses [20–23], though a recent study has argued against this [20–23]. During the investigation of the 2011 outbreaks in Australia, four paramyxoviruses (Hervey virus, Yeppoon virus, Grove virus and Teviot virus) were isolated from bat urine collected from flying fox colonies near to the outbreak areas [12]. Of these three newly isolated bat viruses, three clustered with Asian bat rubulaviruses, while Hervey virus (HerPV) was distinct in being closer related to Achimota virus 2 isolated from bats in Ghana [12]. This further underlines the hypothesis of co-evolution between bats and Paramyxoviruses. Here we report the characterization of HerPV, a proposed member of the genus *Rubulavirus*. HerPV was fully characterized by growth kinetics, genome sequencing and phylogeny. Furthermore, we investigated the serological response to HerPV and henipaviruses of fruit-eating bats inhabiting the distribution range of *Pteropus spp* from Asia to Africa.

## Material and methods

### Ethics

Fieldwork was conducted under the (then) Queensland Department of Employment, Economic Development and Innovation Animal Ethics Committee Permit SA 2011/12/375, the Queensland Environmental Protection Agency Scientific Purposes Permit WISP05810609, the Queensland Department of Environment and Resource Management Scientific Purposes Permit WISP05810609, the New South Wales Office of Environment and Heritage Animal Ethics Committee Permit 120206/02 and the New South Wales Office of Environment and Heritage Scientific Licence SL 100537. The fieldwork comprised the collection of pooled urine samples from under roosting flying foxes by trained and experienced personnel. No animals were captured or sacrificed. Archived samples used in the study were collected under required permits as detailed in the relevant primary publications [24,25].

To characterize HerPV, we examined growth characteristics, in-vitro host range and serological reactivity of bats in the *Pteropus* distribution range to HerPV and Henipaviruses.

### Sample origin: Virus, cell lines and serum samples

HerPV was isolated from three independent urine samples of *Pteropus spp*. collected in Hervey Bay (QLD), Boonah (QLD) and Nambucca Heads, New South Wales (NSW), Australia, as described previously [12]. Isolates ‘Boonah 4’, ‘Hervey 7’ and ‘Boonah 6’ were used in this study. Table 1 lists all cell lines used, including the *Pteropus alecto* cell lines established in our group [26]. The bat sera tested for reactivity to HerPV in this study were samples remaining from previously published studies [24,27] (S1 Table).

**Table 1:** List of cell lines used in this study and summary of results for each cell line.

Cell line	Starting cell concentration TCID_50_	TCID_50_ titer	IIFAT (96-well plate) at HerPV dilution*	Cell type	Species
Vero E6	2.0 x 10^5^/ml	1.5 x 10^5^	n/d	Kidney	Monkey
LoVo	4.0 x 10^5^/ml	2.8 x 10^3^	n/d	Rectal carcinoma	Human
PaSp	1.5 x 10^5^/ml	No TCID	n/d	Primary spleen	Bat
PaHe	2.0 x 10^5^/ml	4.1 x 10^4^	1,00E-04	Primary heart	Bat
PaBr	1.5 x 10^5^/ml	No TCID	n/d	Primary brain	Bat
PaKi	3.0 x 10^5^/ml	6.1 x 10^4^	n/d	Primary kidney	Bat
Equine kidney	4.0 x 10^5^/ml	8.6 x 10^3^	n/d	Primary kidney	Horse
MDCK	1.5 x 10^5^/ml	No TCID	1.0 x 10^2^	Kidney	Dog
Hep2	2.0 x 10^6^/ml	No TCID	1.0 x 10^2^	Cervical carcinoma	Human
CRFK	9.0 x 10^5^/ml	1.9 x 10^4^	1.0 x 10^4^	Kidney	Cat
Bovine kidney	1.0 x 10^6^/ml	4.9 x 10^4^	1.0 x 10^4^	Kidney	Bovine

n/d, not determined

* The numbers in this column are giving the minimal virus concentrations where fluorescence was visible after staining of the fixed TCID50 test.

#### Virus propagation and preparation of stocks

For virus propagation, virus was inoculated onto PaKi or Vero E6 cell monolayers. Cell culture supernatant containing HerPV was harvested up to seven days after incubation, when cells showed significant cytopathic effects (CPE). HerPV was clarified by centrifugation at 2000 rpm for 30 min (Heraeus megafuge 40R, TX400 swinging rotor, 1900 xg) and stored in aliquots at -80°C until use.

#### Virus titration

Growth of HerPV was examined in different host cell lines listed in Table 1. The Tissue Culture Infectious Dose _*50*_ (TCID_50_) was determined for all cell lines by 10-fold dilutions of HerPV stocks with 10 replicates per sample dilution test [28]. All cell lines were treated likewise: Cells were freshly trypsinized, counted and diluted to the aimed concentration in DMEM containing 10% FCS. The respective concentration optimized (90% confluence after 24 hours of incubation) per individual cell line was determined in advance and is shown in Table 1. In a 24-well cell culture plate 10-fold serial dilutions of a freshly thawed HervPV stock were prepared in DMEM containing 2% FCS. 100 μl of each HerPV dilution (from 10^−3^ to 10^−10^) was pipetted to 10 wells of the microtitre plate (96 well), starting from highest dilution to the lowest. Columns 11 and 12 were used as negative controls and contained 100 μl DMEM (+2% FCS) per well. Subsequently, 100 μl of the cell culture dilution was dispensed to each well of the microtiter plate. Plates were incubated at 37°C as described before. The TCID_50_ tests were analyzed after seven days of incubation. Results were obtained by visual recognition of cytopathic effects and calculation of titres by using the calculation by Reed & Muench [27]. Additionally, infected cells in 96-well plates were stained for indirect immunofluorescence assay (IIFA). To prepare 96-well plates with infected cells for IIFAT, the supernatant was removed and the cells were washed with cold PBS and air dried before fixation with methanol (-20°C) for 30 min at 4°C. Cells were dried and prepared plates stored at -20°C for future use.

#### Indirect IFAT (IIFAT) screening using fluorescence microscopy

**Fig 1:**
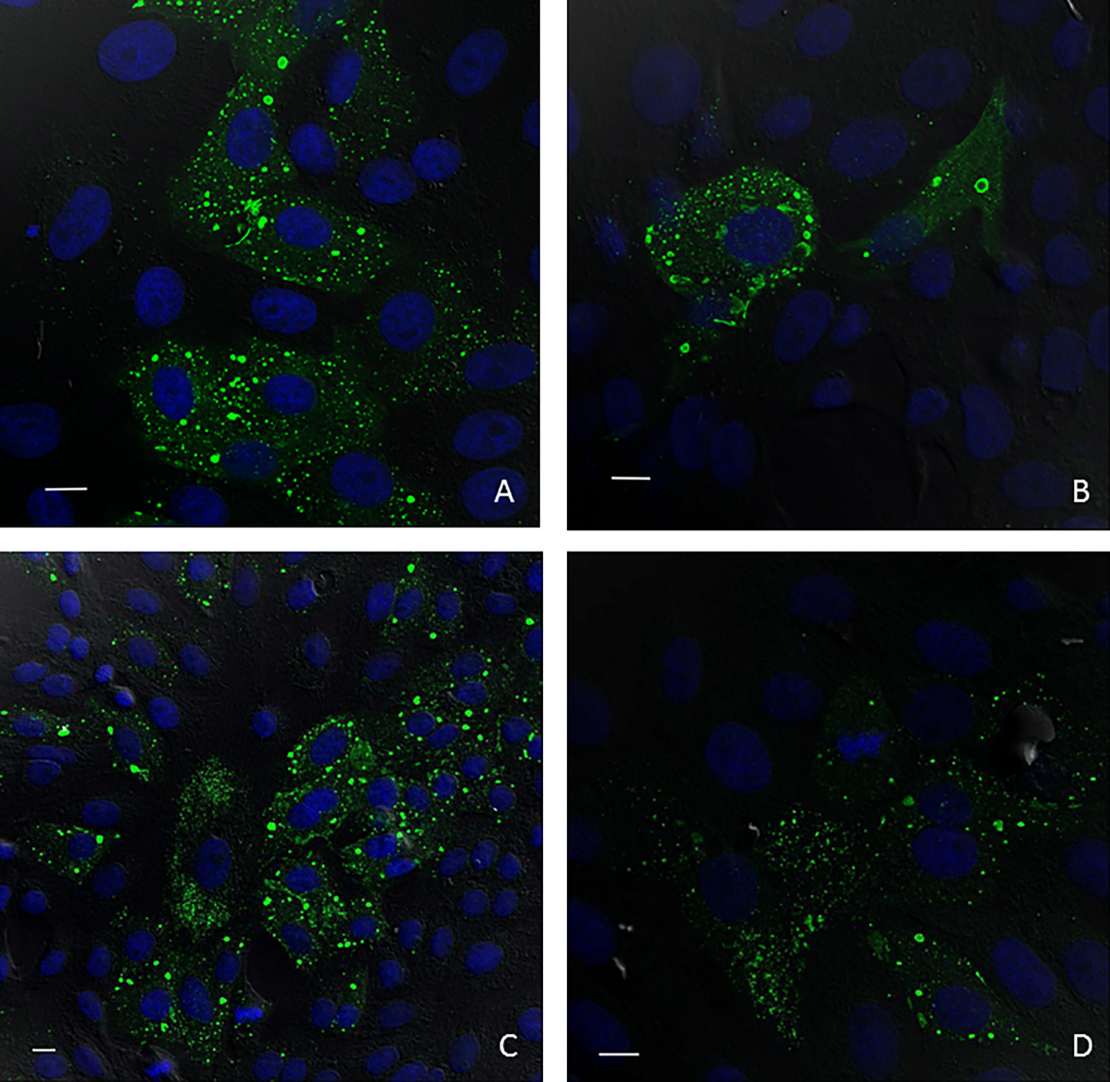
Confocal images of HerPV-infected cells incubated with different sera. (**A**) and (**B**) Examples of IIFAT result with sera from *P*. *poliocephalus* (#9, Bendigo) from Victoria, Australia; (**C**) Rabbit anti-Menangle virus serum; and (**D**) Pig anti-Menangle virus serum. Scale = 10um.

For general determination of the presence of HerPV-reactive IgG antibodies in bat serum or urine samples, IIFAT slides were prepared as follows. Vero E6 cells with a cell concentration of 2.0 x 10^6^ cells/ml were mixed with HerPV at a multiple of infection (MOI) of 1 and seeded onto 8-well chamber slides (Nunc). The slides were incubated under standard cell culture conditions for three days until a visible CPE developed. For fixation, the supernatant was discarded and the monolayers were washed twice with phosphate buffered saline (PBS). Subsequently, wells were overlaid with cold acetone (-20°C) and incubated at 4°C for 20 min. The grids were peeled off and slides air-dried before storage at -20°C until use. Control test slides were similarly prepared. All bat sera were diluted 1:50 in PBS. Each diluted bat serum (80 μl) was inoculated onto one well of a fresh IIFAT slide. As a negative control, one well was inoculated with 80 μl of PBS per slide. Slides were incubated in a humidified chamber at 37°C for 40 min. The slides were washed 3 times in fresh PBS for 5 min to remove excess sera. Slides were air-dried before incubation with conjugate (Alexa 488 labeled protein A (Invitrogen); diluted in PBS) for 45 min as above. Washing was repeated as described. After air-drying, slides were mounted with Fluorescent Mounting Media containing DAPI (Sigma Aldrich) and analyzed using an EVOS fluorescence microscope. IIFAT 96 wells were stained similarly using serum from *P*. *poliocephalus* (#9, Bendigo) (Fig 1A and 1B). Bat urine samples were processed with the same procedure, except for the starting dilution of 1:10.

#### Indirect IFAT (IIFAT) examination using confocal microscopy

For specific sera and the evaluation of cross-reactivity, we used a different IIFAT protocol which is more time consuming but allows for a more detailed observation of effects. Vero cells (8 x 10^4^) were seeded into each well of a 24-well cell culture plate containing 13 mm coverslips (Menzel-Glaser) and incubated overnight. Medium was replaced with 200 μl of HerPV suspension at MOI 1 and MOI 0.1, respectively. After 1 h of incubation under cell culture conditions, the virus suspension was replaced with medium and the plate was incubated overnight. For fixation, the medium was removed and the cells were overlaid with freshly thawed paraformaldehyde (4%) and incubated at room temperature for 50 min. Fixative was removed by washing 3 times with PBS and slides were stored in PBS at 4°C. Negative controls were prepared likewise. For IIFAT, the fixed cells on each coverslip were treated with 1 ml of 0.1% Triton X-100 for 10 min, followed by blocking with 0.5% BSA in PBS for 30 min. Test sera were then diluted in PBS containing 0.5% BSA (1:50) and incubated with the cells for 1 h. Cells were washed 3 times with PBS for 5 min and then incubated with secondary antibody for 1 h (Alexa 488 labeled protein A diluted 1:2,000 in PBS/0.5% BSA). Coverslips were washed twice with PBS for 5 min before rinsing twice with water. Nuclei were labelled with DAPI (Sigma Aldrich) for 10 min, rinsed with water and mounted onto a glass slide for microscopy.

#### Serum Neutralization Tests (SNT)

All serum neutralization assays were carried out in 96 well cell culture plates as previously described [28]. Sera were heat inactivated at 56°C for 30 min. Bat sera, diluted 1:20 to 1:2560 in EMEM, were incubated with an equal volume of HerPV containing 100 TCID_50_ for 30 min prior to the addition of 2 x 10^4^ Vero E6 cells to each well. Negative control plates were prepared without sera. Plates were incubated at 37°C for 3 days under cell culture conditions before assessing visible CPE. To test for cross-neutralization, the following sera were used: anti-Nipah virus (rabbit), anti-Nipah virus (pig), anti-Hendra virus (horse), anti-Tioman virus (rabbit), anti-Menangle virus (pig, infection), anti-Menangle virus (rabbit, hyper-immune) and negative horse serum all at a 1:100 starting dilution.

#### Henipavirus luminex binding assay

All bat sera were also tested using the Henipavirus Luminex binding assay as described by [28] for the presence of antibodies binding to Hendra virus and Nipah virus glycoproteins.

#### NGS and genome assembly

For Next Generation Sequencing (NGS), stock aliquots (clear centrifuged supernatant) were further purified by discontinuous sucrose gradient (36,000 rpm, 90 min, SW41). Virus was collected at the interface (between 50% and 20% sucrose) and extracted with the QIAamp viral RNA Mini Kit (Qiagen). RNA was further purified with QIAquick columns, eluted in 30 μl elution buffer and measured with Nanodrop: RNA was transcribed to cDNA using random primers as described and random PCR was performed as previously described [7]. Subsequently, cDNA was precipitated with the addition of absolute ethanol to a final concentration of 70% to permit sample transfer between laboratories. Samples were prepared for next generation sequencing with the Illumina Nextera XT library preparation kit. Paired-end (PE) 250 bp sequencing was performed on the Illumina MiSeq by Micromon at Monash University, Victoria, Australia. The CLC Genomics Workbench 8.5 software (CLC bio, Aarhus, Denmark) was used for subsequent quality control and secondary analysis of Illumina reads. Briefly, raw reads were trimmed of Nextera XT adapters, primer sequence used for cDNA and PCR amplification as well as ambiguous and low-quality reads (PHRED score ≤ 30). Trimmed data was assembled *de novo* (default settings). Verification of genome ends was performed by read mapping to the *de novo* assembled contig (duplicate reads removed; CLC read mapper default settings). Read-mapped dataset was also used to estimate the P gene RNA editing G insertion frequency by using the CLC Basic Variant Detection tool (default settings except Minimum frequency = 0.01%, Ploidy = 1, Base quality filter = Yes, Relative read direction filter = No).

#### Genome annotation and phylogeny

The HerPV genome sequence (GenBank KU672593) was annotated for comparison using Softberry FGENESV0 (http://www.softberry.com), Geneious Pro R8.1.7 (http://www.geneious.com) and Glimmer [29–31]. For the phylogenetic tree reconstruction, the L-protein coding for the RNA-dependent RNA polymerase was aligned with ClustalW to all related sequences from NCBI GenBank and the ICTV Paramyxovirus type species (6,944 bp, 14 taxa) [32]. The output alignment was quality checked with T-coffee [33]. Prior to tree reconstruction, the evolutionary model was estimated with JModelTest using PHYML likelihood scores in 88 models [34]. The AIC analysis found that the Jules Cantor model matched the dataset best (AIC 52, Nst = 1; prset = equal). Reconstruction was calculated with MrBayes in MCMC chains (1,000,000 replicates, 4 heated chains, burn in 150,000) [35]. The consensus tree was visualized in FigTree (http://tree.bio.ed.ac.uk/software/figtree/). The genetic distances of the whole genomes for 29 viruses (known Paramyxoviruses from bats and the ICTV Paramyxovirus type species) were compared using the distance matrix of Geneious Pro R8.1.7.

## Results

### Spatial data

During the Hendra virus outbreaks in 2011, bat urine was sampled in close proximity to the affected properties [12]. HerPV was first isolated from pooled bat urine samples taken on 20^th^ July 2011 at the Hervey Bay Botanic Gardens, Hervey Bay, QLD, Australia. HerPV was also isolated from pooled bat urine collected on 27^th^ of July 2011 in Nambucca Heads, NSW and on 11^th^ of July 2011 in Boonah, Queensland. Overall, HerPV was isolated from eight samples at three time points and locations. Hendra virus was isolated four times from these same samples.

### Genome characterization

Since the three isolates of HerPV were almost identical based on sequencing of the partial L gene [36] (99.5% identity, unpublished data), only one isolate, ‘Boonah 6’ was subjected to whole genome sequencing using Illumina NGS. A total of 5,484,106 PE reads was obtained, and further trimming for low-quality, ambiguous and adapter sequences resulted in 5,471,118 PE reads with average read length of 133 bp (range 1–251 bp). *De novo* assembly of trimmed PE reads produced 66,685 contigs (N50 = 470) with the largest contig coding for the near complete genome of HerPV, missing only 6 and 8 nt each at the 5’ and 3’ end, respectively. Comparison to the genomes of closely related rubulaviruses Menangle virus (Genbank Accession No. NC_007620) and Tioman virus (Genbank Accession No. NC_004074) revealed a partial match of the 5’ and 3’ genome end (7 nt and 5 nt, respectively) to the “ACCAAGGGGARAAT” sequence conserved at the genome terminal ends of known and proposed members of the genus *Rubulavirus* [37]. Consequently, the HerPV missing genome termini were inferred from the Rubulavirus consensus sequence, resulting in a predicted genome size of 15,186 nt conforming to the rule of six observed for all members of the *Paramyxovirinae* [15].

**Fig 2:**
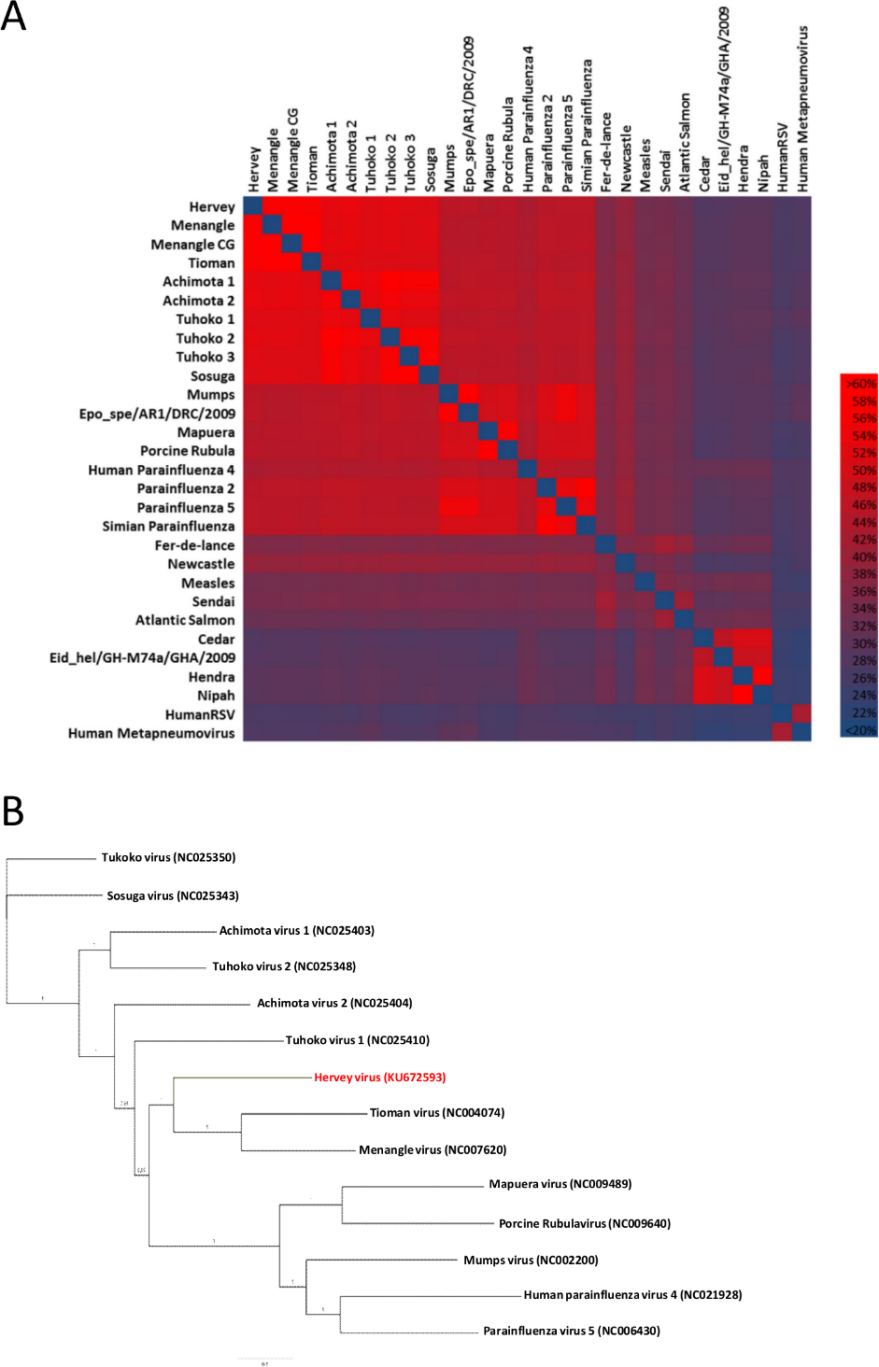
Comparison of HerPV with other Paramyxovirus strains. (**A**) Heatmap of HerPV L protein in comparison to ICTV type species of paramyxoviruses and other paramyxoviruses retrieved from or associated with bats. (Acc. No. KU672593, AF326114, JX112711, AF298895, JX051319, JX051320, GU128080, GU128081, GU128082, KF774436, JN012242, HQ660095, EF095490, BK005918, KF483663, X57559, NC006430, AF052756, AY141760, KP089979, AB016162, M30202, EU156171, JQ001776, HQ660129, AF017149, AF212302, KJ723491,GQ153651) (**B**) Reconstructed phylogenetic tree based on the partial L gene (6,944 nt) of HerPV in comparison to other rubulaviruses. Calculation was performed using MrBayes in MCMC chains (1,000,000 replicates, 4 heated chains, burn in 150,000, JC model). Posterior probabilities are depicted (Acc. No.NC025350, NC025343, NC025403, NC025348, NC025404, NC025410, KU672593, NC004074, NC007620, NC009489, NC002200, NC021928, NC006430).

All CDS and proteins were identified and annotated by softberry FGENESV and confirmed with Glimmer and Blastp of the NCBI nr database. Even though the virus was purified with a sucrose gradient, contaminant viral transcripts were also obtained which facilitated the determination of the P gene RNA editing site. The presence of 1, 2, 3 or 4 G non-templated insertions were found after nt position 2407. Although the total reads of edited transcripts only accounts for 1.5% of the viral sequences obtained, this does however suggest that P, V and W proteins may be expressed by this virus. The genome characteristics of HerPV strongly suggests that it should be placed within the genus *Rubulavirus* (Accession number KU672593). Phylogenetic analysis based on both RNA and deduced amino acid sequences of different genes demonstrated that this new virus is genetically closely related to, but distinct from, another two proposed Asian bat rubulaviruses Tioman virus and Menangle virus (Fig 2(A) and 2(B)).

### Virological characterization

HerPV was inoculated onto different cell lines to determine its TCID_50_ in these cells (Table 1). The highest titres were reached with Vero E6, Bovine kidney, PaKi and PaHe cells. Low titres were recorded for primary Equine kidney, CRFK and LoVo cells. No visible CPE was demonstrated on MDCK, HEp-2, PaBr and PaSp cells, although HEp-2 and MDCK cells showed fluorescence in the subsequent 96-well IIFAT assay (1x10^2^).

### Serological characterization

As a pilot study, IIFAT slides were used to determine cross-reactivity with anti-sera to selected paramyxoviruses (Cedar virus, Hendra virus, Menangle virus, Nipah virus and Tioman virus). No cross-reactivity was observed to Cedar virus (rabbit serum) and Hendra virus (rabbit and horse serum), and very low cross-reactivity was observed to Menangle virus (pig serum), Tioman virus (pig serum) and Nipah virus (rabbit serum). High cross-reactivity was demonstrated solely with Menangle virus (rabbit sera) (Fig 1(C), S2 Table). All anti-sera were tested in a cross-neutralization assay in which no significant cross-neutralization was observed.

#### Prevalence of Anti-HerPV antibodies in bat sera of different geographic origins

**Fig 3:**
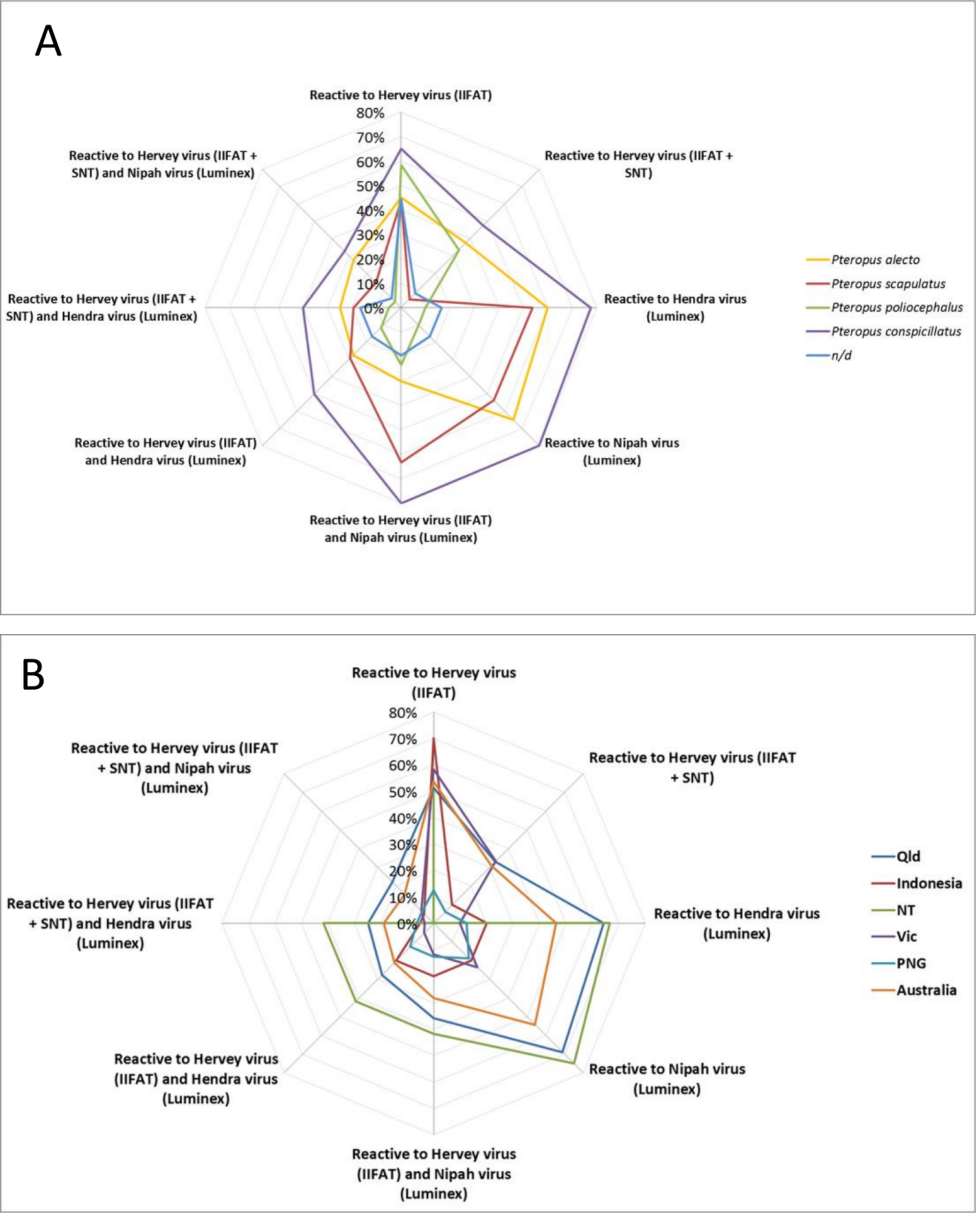
(A). Net plot to visualize correlations found between bat species and reactivity to HerPV in IIFAT, SNT and positive reactivity to Hendra and Nipah viruses. n/d, not determined.(B). Net plot to visualize correlations found between bat origin and reactivity to HerPV in IIFAT, SNT and positive reactivity to Hendra and Nipah viruses. Qld, Queensland; NT, Northern Territory; Vic, Victoria; and PNG, Papua New Guinea.

Overall, 259 bat sera from different locations in Australia, Asia and Africa were screened by IIFAT for the presence of cross-reactive HerPV antibodies. Screening was conducted with sera from *Pteropus alecto* [n = 42], *Pteropus poliocephalus* [n = 60], *Pteropus scapulatus* [n = 41], *Pteropus conspicillatus* [n = 40] and from bats the species of which was not determined (n/d) [n = 36] (S1 Table). All species tested had cross-reactive antibodies to HerPV ranging from 20% (*Eidolon helvum*) to 65% (*Pteropus conspicillatus*) (Table 2, Fig 3 (A)). One-way and two-way ANOVA calculations resulted in significant differences (*) in the detection of reactive antibodies in *Eidolon helvum* and *Pteropus poliocephalus* (S3 Table).

**Table 2:** Summary of serological results based on species.

* *	*P*. *ale*	*P*. *sca*	*P*. *con*	*P*. *pol*	*E*. *hel*	n/d
Bats tested	42	41	40	60	20	36
Positive Hervey IIFAT	18 (43%)	18 (44%)	26 (65%)	35 (58%)	4 (20%)	16 (44%)
Positive Hervey IIFAT + SNT	15 (36%)	2 (5%)	19 (48%)	20 (33%)	0 (0%)	3 (8%)
Positive Henipa (Luminex)	26 (65%)	26 (63%)	32 (80%)	14 (23%)	n/d	7
Positive Hervey + Henipa	11 (28%)	13 (33%)	13 (33%)	2 (3%)	n/d	2 (6%)

*P*. *ale*, *Pteropus alecto*; *P*. *sca*, *Pteropus scapulatus*; *P*. *con*, *Pteropus conspicillatus*; *P*. *pol*, *Pteropus poliocephalus*; positive Henipa, Luminex assay reactive to Hendra and Nipah virus, respectively; n/d, species unknown.

Bats were sampled in differing locations in Australia (in Queensland, Torres strait [n = 18], Brisbane [n = 17], East Brisbane [n = 8], Gordonvale [n = 40], Hervey Bay [n = 14], Gladstone [n = 29]; in New South Wales, Nambucca Heads [n = 5]; in Victoria, Bendigo [n = 8], Geelong [n = 52]; in Northern Territory; [n = 12]) and in Indonesia, Timor [n = 20], Papua New Guinea (PNG) [n = 16] and Africa (Tanzania [n = 8] and the Gulf of Guinea [n = 12]). Antibodies reacting to HerPV were detected in bat sera from all countries examined. The prevalence ranged from 13% (PNG) to 70% (Indonesia) (Table 3, Fig 3(B)). One-way and two-way ANOVA calculations did not reveal any significant differences in the detection of reactive antibodies in bat sera from differing locations.

**Table 3:** Summary of results based on location.

	Australia	Qld	Vic	NT	PNG	IDN	Africa
Bats tested	183	111	60	12	16	20	20
Positive Hervey IIFAT	97 (53%)	56 (50%)	35 (58%)	6 (50%)	2 (13%)	14 (70%)	4 (20%)
Positive Hervey IIFAT + SNT	56 (31%)	36 (32%)	20 (33%)	0 (0%)	1 (6%)	3 (15%)	0 (0%)
Positive Henipa (Luminex)	51 (54%)	39 (69%)	7 (23%)	5 (75%)	2 (19%)	4 (20%)	n/d
Positive Hervey + Henipa	28 (15%)	24 (22%)	4 (7%)	0 (0%)	1	1	n/d

positive Henipa, Luminex assay reactive to Hendra and Nipah virus, respectively; n/d, not determined; Qld, Queensland; Vic, Victoria; NT, Northern Territory; IDN, Indonesia.

All bat sera found reactive in IIFAT screening were subjected to serum neutralization tests. The percentage of bat sera causing neutralization of HerPV ranged from 0% (*Eidolon helvum*) to 48% (*Pteropus conspicillatus)*. One-way ANOVA calculations did not reveal any significant differences in the ratio between detection of reactive antibodies and their ability to neutralize HerPV in bat sera from differing species.

The percentage of neutralizing sera ranged from 0% (Africa, Northern Territory) to 33% (Victoria). One-way ANOVA calculations did result in significant differences in the detection of reactive antibodies in bat sera from Australia in comparison to Indonesia (**) and Africa (**) (S3 Table).

#### Serological investigation of co-infection with henipaviruses

Based on the co-shedding of HerPV and Hendra virus observed in the isolation studies, we were interested in investigating the co-infection at population level by examining antibodies to henipaviruses in the bat serum collection in this study. Bat sera samples (n = 239) were tested for the presence of henipavirus-binding antibodies by the Luminex binding test. There were variations in the percentage of Hendra virus positives depending on the origin of the sera, ranging between 10% in Victoria to 67% in the Northern Territory and 64% in Queensland, respectively; reactivity to Nipah virus had a similar range with 19% in Papua New Guinea and 75% in the Northern Territory. We found the highest number of positive sera from *Pteropus conspicillatus* (78–80%), while the lowest number was observed in *Pteropus poliocephalus* (10%) (Tables 2 and 3 and S1, Fig 3(A) and 3(B)).

## Discussion

Hervey virus (HerPV) is a novel bat paramyxovirus named after the place of its first isolation, Hervey Bay in QLD, Australia. The virus is similar in genome organization, length and protein annotation to other proposed members of the genus *Rubulavirus* isolated from bats. The initial phylogenetic tree, comprising the short HerPV RdRP sequence, showed HerPV clustering to Archimota virus 2 from Africa [12]. In contrast, our new analysis that was based on the whole RdRP ORF (6,944 nt) showed that HerPV is closest related to Menangle virus and Tioman virus. However, the highest genetic similarity observed (to Menangle virus and Tioman virus) is around 60% or less (as shown by the heatmap in Fig 2(A)), supporting it as a new species, most probably in the genus *Rubulavirus*. This is further supported by the low antibody cross-reactivity to Menangle virus or Tioman virus, the lack of cross-neutralization and the phylogenetic analysis demonstrating that HerPV is distinct from the other bat-derived rubulaviruses. The donor dependent nature of the sera, coupled with different methods of antibody production (immunization with recombinant protein for the rabbits and experimental infection with live virus for the pigs) would most likely explain the variability in reactions observed with the anti-Menangle virus sera from pigs and rabbits. The ability of HerPV to grow in different cell cultures indicates a potential opportunistic broad host range for this novel virus, as with Hendra virus. Of all cell lines tested, it was only primary bat brain and spleen cells in which no replication could be detected.

The Hendra virus outbreaks in 2011 were exceptional in many ways. Distinct outbreaks were more numerous than in the other years. Outbreaks were located further south and west than previously reported, and 17 of them were in close proximity to each other (40–700 km). Bat urine was sampled from bat colonies adjacent to a number of outbreak sites. Hendra virus was successfully isolated from a number of these urine samples, demonstrating that Hendra virus was indeed circulating in these colonies. Along with a number of other paramyxoviruses, Hendra virus was detected in urine from all three locations where HerPV was isolated. HerPV was detected in under-roost urine samples from three different locations approximately 700 km apart over a 16-day period: in Boonah on 11 July 2011 and 26 July 2011, in Hervey Bay on 20 July 2011 and in Nambucca Heads on 27 July 2011.

To investigate the diversity of HerPV in pteropid bats, we tested reactivity of sera from a variety of pteropid species to HerPV by IIFAT and the sera positive by IIFAT were then subjected to HerPV SNT. All four Australian pteropid bat species showed similar high reactivity to HerPV at 43–65% (Fig 3(A) and 3(B). However, the ability to neutralize HerPV was lower in *Pteropus scapulatus*.

*Eidolon Helvum* bats from Africa showed a low percentage of reactivity to HerPV by IIFAT and none had the ability to neutralize the virus. Not surprisingly, viruses circulating in African bats may be less closely related. This is supported by the genetic diversity and spatial distance of fruit-eating bats between Africa and Australasia. However, our findings suggest the presence of yet unknown rubulaviruses in African bats, supporting the theory of the world-wide distribution of paramyxoviruses in bats.

Pteropid bats from PNG and Indonesia displayed varying reactivity with low neutralization of HerPV. Bats from PNG showed a higher percentage of reactivity to Nipah virus, while bats from Indonesia displayed a higher percentage of reactivity to Hendra virus and HerPV. These results support the theory of competitive exclusion previously described for Hendra virus and Nipah virus [25]. Because it is not a BSL-4 agent, investigation of HerPV prevalence and ecology is much less constrained than a similar study on Henipaviruses, making it a potentially useful sentinel agent.

Taking all results into account, we conclude that co-shedding activity of Hendra virus and HerPV might reflect similar ecological patterns. Consequently, the factors influencing increased shedding of Hendra virus may also increase that of HerPV. Moreover, further studies should investigate the possibility of co-infections of Hendra virus and HerPV as also indicated by the *in vitro* host range of HerPV. As a first step, archived samples from previous Hendra virus outbreaks could be screened for the presence of HerPV antibodies and RNA. Studies focusing on the ecological dynamics of HerPV may also shed light on dynamics of Hendra virus infections.

## Supporting information

S1 Table Bat samples per species and location. (DOCX)

S2 Table Cross-reactivity and cross-neutralization of HerPV with sera against selected paramyxoviruses. (DOCX)

S3 Table 1-way Anova, repeated measures, Bonferroni post test, bat species. (DOCX)

S4 Table 1-way Anova, repeated measures, Bonferroni post test, origin of bats. (DOCX)
