# Supplementary material for: Hervey virus: Study on co-circulation with Henipaviruses in Pteropid bats within their distribution range from Australia to Africa
Source: PLoS One. 2018 Feb 1;13(2):e0191933. doi: 10.1371/journal.pone.0191933 (PMC5794109; doi:10.1371/journal.pone.0191933)
Supplement: S1 Table — (DOCX) [file pone.0191933.s001.docx]

Supporting information

S1 Table . Bat samples per species and location.

|  | ***Pteropus alecto*** | **n/d** | ***Pteropus scapulatus*** | ***Pteropus conspicillatus*** | ***Eidolon helvum*** | ***Pteropus poliocephalus*** | **Total** |
| --- | --- | --- | --- | --- | --- | --- | --- |
| **QLD** | 42 | 20 | 29 | 40 | 0 | 0 | 131 |
| **VIC** | 0 | 0 | 0 | 0 | 0 | 60 | 60 |
| **NT** | 0 | 0 | 12 | 0 | 0 | 0 | 12 |
| **Asia** | 0 | 36 | 0 | 0 | 0 | 0 | 36 |
| **Africa** | 0 | 0 | 0 | 0 | 20 | 0 | 20 |
| **Total** | 42 | 36 sera (+20 urine) | 41 | 40 | 20 | 60 | 259 |

Abbreviations: QLD, Queensland; VIC, Victoria; NT, Northern Territory; n/d, species unknown.
