# Supplementary material for: Hervey virus: Study on co-circulation with Henipaviruses in Pteropid bats within their distribution range from Australia to Africa
Source: PLoS One. 2018 Feb 1;13(2):e0191933. doi: 10.1371/journal.pone.0191933 (PMC5794109; doi:10.1371/journal.pone.0191933)
Supplement: S2 Table — (DOCX) [file pone.0191933.s002.docx]

Supporting information

S2 Table. Cross-reactivity and cross-neutralization of HerPV with sera against selected paramyxoviruses.

| **Virus** | **Animal** | **Dilution** | **Result IFA** | **Result SNT** |
| --- | --- | --- | --- | --- |
| **Cedar** | Ferret | 1:100 | negative | negative |
| **Cedar** | Rabbit | 1:100 | negative | negative |
| **Hendra** | Horse | 1:100 | negative | negative* |
| **Hendra** | Rabbit | 1:100 | negative | negative |
| **Menangle** | Pig 5 | 1:100 | negative | negative |
| **Menangle** | Pig 1 | 1:100 | + | negative |
| **Menangle** | Rabbit | 1:100 | +++ | negative |
| **Menangle** | Rabbit „4“ | 1:100 | negative | negative |
| **Nipah** | Pig | 1:100 | negative | negative* |
| **Nipah** | Rabbit | 1:100 | + | negative* |
| **Tioman** | Pig | 1:100 | + | negative |
| **Tioman** | Rabbit | 1:100 | negative | negative |
| **Normal Horse** | Horse | 1:100 | negative | negative |

*Weak inhibition observed at low dilution (1:20) which was not sustained with further dilutions.
