# Supplementary material for: Hervey virus: Study on co-circulation with Henipaviruses in Pteropid bats within their distribution range from Australia to Africa
Source: PLoS One. 2018 Feb 1;13(2):e0191933. doi: 10.1371/journal.pone.0191933 (PMC5794109; doi:10.1371/journal.pone.0191933)
Supplement: S3 Table — (DOCX) [file pone.0191933.s003.docx]

Supporting information

S3 Table. 1-way Anova, repeated measures, Bonferroni post test, bat species.

| **Bonferroni's Multiple Comparison Test** | **Mean diff,** | **t** | **Significant? P < 0,1?** | **Summary** | **90% CI of diff** |
| --- | --- | --- | --- | --- | --- |
| black vs red | 0.5000 | 0.1406 | No | ns | -15.88 to 16.88 |
| black vs spec | -3.000 | 0.8435 | No | ns | -19.38 to 13.38 |
| black vs grey | -17.50 | 4.920 | Yes | ns | -33.88 to -1.125 |
| black vs Eidolon | 18.00 | 5.061 | Yes | ns | 1.625 to 34.38 |
| red vs spec | -3.500 | 0.9841 | No | ns | -19.88 to 12.88 |
| red vs grey | -18.00 | 5.061 | Yes | ns | -34.38 to -1.625 |
| red vs Eidolon | 17.50 | 4.920 | Yes | ns | 1.125 to 33.88 |
| spec vs grey | -14,50 | 4.077 | No | ns | -30.88 to 1.875 |
| spec vs Eidolon | 21,00 | 5.904 | Yes | * | 4.625 to 37.38 |
| grey vs Eidolon | 35,50 | 9.981 | Yes | ** | 19.12 to 51.88 |
