# Supplementary material for: Hervey virus: Study on co-circulation with Henipaviruses in Pteropid bats within their distribution range from Australia to Africa
Source: PLoS One. 2018 Feb 1;13(2):e0191933. doi: 10.1371/journal.pone.0191933 (PMC5794109; doi:10.1371/journal.pone.0191933)
Supplement: S4 Table — (DOCX) [file pone.0191933.s004.docx]

Supporting information

S4 Table. 1-way Anova, repeated measures, Bonferroni post test, origin of bats.

| **Bonferroni's Multiple Comparison Test** | **Mean diff,** | **t** | **Significant? P < 0,05?** | **Summary** | **95% CI of diff** |
| --- | --- | --- | --- | --- | --- |
| Australia vs Qld | 56,50 | 2,038 | No | ns | -82,95 to 195,9 |
| Australia vs Vic | 102,5 | 3,697 | No | ns | -36,95 to 241,9 |
| Australia vs NT | 141,0 | 5,086 | Yes | * | 1,554 to 280,4 |
| Australia vs PNG | 141,0 | 5,086 | Yes | * | 1,554 to 280,4 |
| Australia vs Indonesia | 133,0 | 4,797 | No | ns | -6,446 to 272,4 |
| Australia vs Africa | 138,0 | 4,978 | No | ns | -1,446 to 277,4 |
| Qld vs Vic | 46,00 | 1,659 | No | ns | -93,45 to 185,4 |
| Qld vs NT | 84,50 | 3,048 | No | ns | -54,95 to 223,9 |
| Qld vs PNG | 84,50 | 3,048 | No | ns | -54,95 to 223,9 |
| Qld vs Indonesia | 76,50 | 2,759 | No | ns | -62,95 to 215,9 |
| Qld vs Africa | 81,50 | 2,940 | No | ns | -57,95 to 220,9 |
| Vic vs NT | 38,50 | 1,389 | No | ns | -100,9 to 177,9 |
| Vic vs PNG | 38,50 | 1,389 | No | ns | -100,9 to 177,9 |
| Vic vs Indonesia | 30,50 | 1,100 | No | ns | -108,9 to 169,9 |
| Vic vs Africa | 35,50 | 1,280 | No | ns | -103,9 to 174,9 |
| NT vs PNG | 0,0 | 0,0 | No | ns | -139,4 to 139,4 |
| NT vs Indonesia | -8,000 | 0,2886 | No | ns | -147,4 to 131,4 |
| NT vs Africa | -3,000 | 0,1082 | No | ns | -142,4 to 136,4 |
| PNG vs Indonesia | -8,000 | 0,2886 | No | ns | -147,4 to 131,4 |
| PNG vs Africa | -3,000 | 0,1082 | No | ns | -142,4 to 136,4 |
| Indonesia vs Africa | 5,000 | 0,1803 | No | ns | -134,4 to 144,4 |
